# Quantitative Evaluation of Regulatory Indicators for Brominated Haloacetic Acids in Drinking Water

**DOI:** 10.1021/acs.est.4c10202

**Published:** 2025-02-25

**Authors:** Kirin Emlet Furst

**Affiliations:** † Sid and Reva Dewberry Department of Civil, Environmental & Infrastructure Engineering, George Mason University, Fairfax, Virginia 22030, United States; ‡ Occoquan Watershed Monitoring Laboratory, The Charles E. Via, Jr. Department of Civil and Environmental Engineering, Virginia Tech, Manassas, Virginia 22152, United States

**Keywords:** disinfection byproducts (DBPs), drinking water, haloacetic acids (HAAs), bromide, policy analysis, regulations

## Abstract

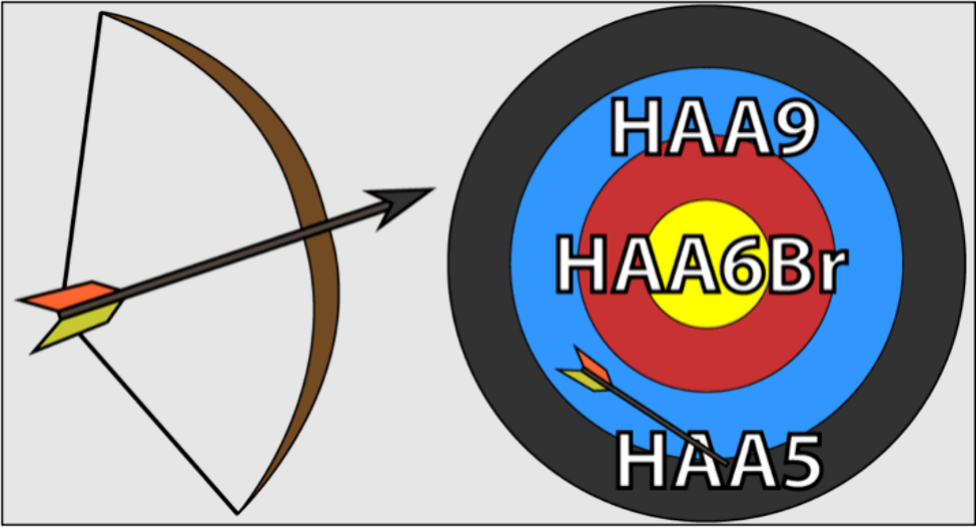

Drinking water regulations often use indicators to represent risk associated with broader contaminant groups. To evaluate the efficacy of indicators, a quantitative approach is needed that aligns with the regulatory framework, in which a benchmark value represents an unacceptably high level of a contaminant or contaminant class. This policy microsimulation study develops such an approach in the context of potential regulatory revisions to address brominated HAAs, a class of disinfection byproducts. Likely scenarios include a limit on the sum of nine brominated and chlorinated HAAs (HAA9), on bromide, or on the sum of six brominated HAAs (HAA6Br). The probability of each potential regulatory indicator co-occurring with a high level of HAA6Br was quantified using logistic models. The HAA9 and bromide indicators both performed poorly, with no better than a ∼1 in 4 chance of identifying equivalently high levels of HAA6Br. Furthermore, high false positive rates (>75%) would implicate a substantial number of water systems that do not have high HAA6Br levels. This study reveals the trade-off implicit in the use of regulatory indicators, in which precision (fewer false positives) must be sacrificed to achieve greater coverage (more true positives). The methodology and findings have broad implications for evaluating indicator classes in drinking water policy and research.

## Abstract 2

A prospective microsimulation policy analysis presents a quantitative method for evaluating regulatory indicators to limit brominated haloacetic acids, a toxicologically important class of disinfection byproducts, in drinking water.

## Introduction

With over 700 organic disinfection byproducts (DBPs) discovered,^1^ there are too many to routinely monitor and regulate. National regulations typically target a subset of trihalomethanes (THMs) and haloacetic acids (HAAs) with the idea that they serve as indicators of the larger DBP mixture.^2,3^ Of the nine chloro- and bromo- HAAs (HAA9), a subset of five (HAA5) are regulated in the U.S., including only two brominated species.^4^ In many countries, none of the six brominated HAAs (HAA6Br) are regulated,^3^ despite findings that five of these are more toxic than the chlorinated species.^5−10^ The U.S. National Toxicology Program found sufficient evidence of human carcinogenicity for only two members of HAA5 (dichloro- and dibromoacetic acid), compared to all four unregulated brominated HAAs (bromochloro-, bromodichloro-, dibromochloro-, and tribromoacetic acid).^11^ Though epidemiologic research has focused on trihalomethanes (THMs), stronger associations have been found with exposure to brominated species than with chloroform for bladder cancer^12^ and reduction in mean birth weight.^13^ This aligns with *in vitro* findings that most brominated DBPs are more toxic than their chlorinated analogues.^6^ The sole epidemiologic study to investigate an unregulated brominated HAA found an association between bromochloroacetic acid exposure and increased colon cancer risk in women,^14^ which was corroborated by an *in vivo* study finding increased tumor occurrence in the large intestine of rats.^7^ A study of U.S. water systems estimated that HAA6Br is cumulatively responsible for 2- to 4-times more cancer cases than HAA5.^15^ Thus, regulations that successfully reduce the highest brominated HAA exposures could have substantial public health benefits, as long as adequate disinfection is not compromised.

The HAA5 Maximum Contaminant Level (MCL) has been identified by U.S. Environmental Protection Agency (EPA) as a candidate for revision in order to address the unregulated brominated HAAs.^16,17^ There is reason to suspect that the HAA5 MCL does not protect against excessive levels of brominated HAAs. The two groups would have to exhibit similar trends across water systems,^18^ while the effect of bromide on HAA speciation suggests the opposite may be true. At low bromide levels, the dominant species are the three chlorine-only HAAs which are addressed by the HAA5 MCL. Moderate bromide levels favor the three mixed bromine/chlorine-HAAs, all unregulated.^19,20^ The highest bromide levels favor the three bromine-only HAAs, bromo- and dibromoacetic acid (regulated) and tribromoacetic acid (unregulated).^19^ Therefore, as bromide concentrations increase, HAA speciation shifts from 100% regulated, to mostly unregulated, to a mix of regulated and unregulated species. Thus, water systems with elevated bromide concentrations can have excessive brominated HAA levels while complying with the HAA5 MCL of 60 μg/L.

What regulatory approach will best address excessive brominated HAA levels in drinking water? The assumption is that EPA will replace HAA5 with HAA9 as the regulated group.^21^ An HAA9 MCL would likely be set at a high concentration (72–77 μg/L) to avoid excessive economic impact to water systems.^21^ For an HAA9 MCL to successfully address high brominated HAA levels, two things must be true: First, the majority of high brominated HAA levels must coincide with exceedances of the HAA9 MCL; it is not clear that this is true. Second, steps taken by utilities to comply with an HAA9 MCL must mitigate brominated HAAs as well as chlorinated HAAs, which may not be the case. THM bromine incorporation factors (BIFs) increased across large U.S. water systems following the Stage 2 DBP Rules, indicating that stricter limits on THMs and HAA5 may have been more effective at limiting chloroform than Br-THMs.^22^ As THM and HAA BIFs are closely related, brominated HAA levels likely increased as well.^23^ One explanation is increased uptake of EPA-designated Best Available Treatments (BATs) for DBP precursor control.^24^ For example, granular activated carbon (GAC) with enhanced coagulation or enhanced softening removes a substantial portion of total organic carbon (TOC) but not bromide, thereby increasing the bromide/TOC ratio and consequently, BIFs.^25,26^ Increased uptake of chloramination could also explain an increase in bromine incorporation in some cases, e.g. for dihalogenated acetic acids in low pH waters.^27^ An alternate explanation for the observed increase in BIFs is rising bromide levels due to industrial discharges^28^ or seawater intrusion,^29^ though these phenomena only affect specific watersheds.^30^ In any case, HAA9 levels may not be sufficiently representative of brominated HAA levels to be an appropriate regulatory indicator.

Another potential approach to limit brominated HAA levels is to regulate bromide with an MCL or treatment technique. Bromide could be an effective indicator because it plays a causal role in the formation of brominated HAAs. Furthermore, bromide levels may relate directly to health risk. For example, a 50 μg/L increase in source water bromide was associated with increased THM levels corresponding to a 10^–4^ to 10^–3^ excess lifetime bladder cancer risk.^31^ Regulating bromide could have the additional benefit of limiting other brominated DBPs of concern. However, bromide removal requires advanced treatment technologies, and is notably challenging and costly to implement.^32,33^ The remaining option for addressing brominated HAAs is to limit them directly, either with MCLs on individual species or as a class (e.g., HAA6Br). HAA6Br was included along with HAA5 and HAA9 in EPA’s Fourth Unregulated Contaminant Monitoring Rule (UCMR4), while individual HAA concentrations were omitted. This signals that EPA is likely to follow the precedent of regulating organic DBPs by class instead of as individual contaminants, and that HAA6Br is a viable regulatory target.^34^

Previous work evaluating DBP regulatory indicators has largely relied on simple correlation analyses to evaluate their efficacy.^3,35−37^ However, correlation analysis is not informative in the context of drinking water regulatory and risk assessment frameworks, in which a single benchmark value of an indicator represents an unacceptably high level of risk. The primary outcome of a correlation analysis is a correlation coefficient (e.g., Pearson’s *r*), which does not have an objective interpretation as applied to this regulatory framework. For example, one study characterized an *r* of ∼0.5 between two DBP groups as strong,^35^ while another concluded that it is unacceptably weak.^3^ Thus, a new approach for indicator analysis is needed to directly align with the regulatory framework for contaminants in drinking water. Such an approach is suggested by Furst et al., who used logistic regression to calculate the bias incurred by using THMs as an exposure indicator for haloacetonitriles in epidemiologic research.^18^ This study found that the THM indicator could systematically underestimate high levels of more toxic DBPs by a factor of 2.3 across U.S. water systems. This approach can be adapted to evaluate the efficacy of regulatory indicators.

This study proposes a novel analytical approach for regulatory indicator evaluation through a series of microsimulations using the UCMR4 dataset (2018–2020).^34^ Four likely scenarios for controlling brominated HAAs are considered: 1) The HAA5 MCL remains in place. 2) The HAA5 MCL is replaced by an HAA9 MCL. 3) A bromide limit is implemented in addition to the current HAA5 MCL. 4) An MCL is implemented for HAA6Br in addition to the current HAA5 MCL. Logistic regression is used to quantify the efficacy of each regulatory target as an indicator for high HAA6Br levels. The initial impacts of each policy scenario are estimated, including the percentage of systems that may be noncompliant, and how accurately the systems with high brominated HAA levels are targeted. Prospective policy analyses are crucial for ensuring that policies are well-designed before they are enacted, thereby avoiding the waste of time and resources. The findings of this study provide insight into which scenarios within the current EPA regulatory paradigm will successfully target brominated HAAs as a class. Finally, future research needs are discussed, particularly the need for health-based advisory levels for individual brominated HAAs to ensure that future policy sufficiently addresses exposure risk.

## Methods

### Data Acquisition and Processing

UCMR4 data was acquired from U.S. EPA’s Web site (https://www.epa.gov/dwucmr/occurrence-data-unregulated-contaminant-monitoring-rule#4) and processed and analyzed in Python. All PWS serving over 10,000 people that are subject to the Disinfection/Disinfection Byproducts Rules (D/DBPRs) were required to sample for HAAs under UCMR4. EPA defined these as “Large” PWS for UCMR4. In addition, a representative sample of 800 small PWS subject to the Disinfection and Disinfection Byproduct Rules (D/DBPRs) were required to sample for HAAs under UCMR4. The small PWS were selected from the population of community water systems and nontransient, noncommunity water systems following methodology developed by EPA (2001).^38^ HAAs were measured by EPA Method 552.3,^39^ and concentrations were summed by class before publication such that individual HAA concentrations were not available for this study. Sixty-eight sample records missing HAA entries were removed, yielding 63,427 complete records representing 4,924 U.S. PWS, which reported between 1 and 88 records each.

For each group of HAAs, if all species were below their method reporting limits (MRLs), the sum was reported as 0 μg/L. UCMR4 HAA MRLs were not reported, yet all three HAA groups had minimum (nonzero) values of 0.2 μg/L,^34^ so <MRL entries were replaced with 0.2 μg/L. The effect of this replacement on study outcomes is negligible (Text S3). The regulatory framework for DBPs applies the MCLs to the Locational Running Annual Average (LRAA) rather than the individual measured concentrations.^4^ LRAA concentrations were calculated for each HAA group as the average measurement for samples taken at a monitoring location during the previous calendar quarters (up to four) and used for all analyses.

PWS characteristics reported in UCMR4 include size, source water type, and disinfectant type. Source water type was reported as surface water, groundwater, groundwater under the influence, or mixed surface and groundwater. The number of PWS and summary statistics for each HAA group by PWS size and source water type are summarized in Table S1. The systematic effects of repeated measures (i.e., multiple samples from each PWS) and categorical variables (size, source water type, season, and disinfectant type) on mean levels of each HAA group were evaluated with multilevel regression models following the methods of Furst et al. (Text S1).^18^

### Precursor Data Screening

All UCMR4 PWS were required to report source water bromide and TOC levels except consecutive systems. PWS utilizing multiple water sources reported precursor levels for each; in lieu of influent flow, source precursor data were averaged by sampling date with the assumption each source contributed equally. This yielded 39,924 records with corresponding bromide and TOC, representing 3,431 PWS. Forty-one percent of bromide and 25% of TOC results were below the MRLs (20 μg/L and 1 mg/L, respectively) resulting in non-normal, nonlog-normal distributions. For each sampling event, PWS reported one source water bromide measurement corresponding to multiple HAA samples. Therefore, for each HAA group, the maximum LRAA concentration corresponding to a single bromide result for each sampling event for each PWS was used in all analyses involving bromide.

Source water precursors removed prior to disinfection are not available to form HAAs. TOC is significantly removed by conventional filtration and other treatment processes; thus, TOC was only used to investigate the distribution of source water bromide/TOC ratios by source type (Figure S1). Of treatment processes currently implemented at full-scale, only high pressure membrane processes, particularly reverse osmosis (RO), have achieved 2-log removal of bromide in real source waters.^33,40^ UCMR4 did not distinguish between RO and other membrane filtration processes. Thus, all systems that reported using membrane filtration (307 PWS, 4,042 records) were excluded for analyses involving bromide. Inspection of PWS with bromide levels greater than 1 mg/L revealed that some of these systems likely did use membrane filtration (Text S2). Thus, systems reporting bromide >1 mg/L were excluded from analyses, and a sensitivity analysis found that the effect of this exclusion on the bromide indicator analyses was unimportant (Text S2). The final bromide data set consisted of 8,647 samples from 3,164 PWS.

To accommodate the nonlog normal, highly skewed bromide data for trend analyses, bromide concentrations were binned into six levels, with level 1 containing all < MRL results, and the remaining data divided into 5 levels of equal size (Table S3). Multilevel regression models were developed for each HAA group to evaluate the relationship with bromide level while controlling for the effect of clustering by PWS and differences between source water type, disinfectant, and season (Text S2).

### Indicator Model Development

Logistic regression was identified as the best available model for the analysis of drinking water regulatory indicators by applying three criteria: 1) The output provides a direct, quantitative answer to questions such as, “what is the probability that a high level of a regulatory indicator co-occurs with a high level of a target contaminant?” 2) The model accommodates drinking water data consisting of hierarchically clustered, non-normally distributed continuous and binary variables. 3) The model is readily implemented by environmental policy researchers without expertise in statistics. Logistic regression is a Generalized Linear Model in which the link function is the logit function, and is the most widely used model for analysis of binary outcome data in relevant fields such as toxicology.^41^

Logistic models were developed following methods described by Furst et al.^18^ Briefly, concentrations of HAA5, HAA9, or bromide were converted to binary indicator variables by comparison to a benchmark concentration (a limit), with concentrations less than or equal to the benchmark assigned “0” and concentrations greater than the benchmark assigned “1”. These binary indicators were regressed against binary or continuous HAA6Br concentrations using logistic models to determine the probability of an indicator coinciding with a high level of HAA6Br. Logistic regression coefficients are the log odds, which can be converted to probability by eq 1, where *x* is the logistic regression coefficient and P(*Y*) is the probability that outcome *Y* is equal to 1.
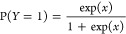
1

To the extent possible, logistic models were defined to account for clustering of data by PWS to avoid the underestimation of errors associated with repeated measures. Assumptions relevant for logistic models are discussed and validated in Text S3, namely 1) the independence of observations, 2) the effect of outliers, and 3) the linearity of independent variables and log odds. All logistic model results presented in the following text are significant to at least the *p* < 0.001 level.

The number of PWS that could be in violation of potential regulatory limits was also calculated for each scenario. In the following discussion, “UCMR4 PWS” refers to all PWS that reported data in the UCMR4 database, and “implicated PWS” refers to the subset of UCMR4 PWS with at least one exceedance of the prospective limit.

## Results and Discussion

The final data set contained 63,427 samples from 4,924 unique PWS. On a mass concentration basis, HAA5 levels were higher than HAA6Br in most samples (Table 1). But HAA6Br did contribute significant mass to HAA9 in many samples, with a median contribution of 30% (mean of 37%) across the data set (Table 1). The four unregulated brominated HAAs comprised greater than half of the mass of HAA6Br in 89% of samples, and 100% of the HAA6Br mass balance in 5.4% of samples. Thus, the majority of HAA6Br mass represents unregulated HAAs not targeted by the HAA5 MCL. Hereafter, all concentrations discussed are LRAA concentrations.

**Table 1:** HAA Group Definitions with UCMR4 Summary Statistics (63,427 Samples)^a^

			Concentration (μg/L)			% mass of HAA9^b^		
Class	Species	Samples ≤ MRL	Mean	Med.	Max.	Mean	Med.	Max.
**HAA9**	CAA, DCAA, TCAA, BAA, DBAA, BCAA, BDCAA, DBCAA, TBAA	3.0%	24.9	22.4	654	NA	NA	NA
**HAA5**	CAA, DCAA, TCAA, BAA, DBAA	3.3%	19.0	15.6	465	72.7	74.7	100
**HAA6Br**	BAA, DBAA, BCAA, BDCAA, DBCAA, TBAA	5.5%	7.2	5.4	446	36.7	29.9	100
**HAA4Br**	BCAA, BDCAA, DBCAA, TBAA	7.1%	6.0	4.7	388	27.3	25.3	100

a *Table key*: MRL: Method Reporting Limit, med.: median, max.: maximum, CAA: chloroacetic acid; DCAA: dichloroacetic acid, TCAA: trichloroacetic acid, BAA: bromoacetic acid, DBAA: dibromoacetic acid, TBAA: tribromoacetic acid, BCAA: bromochloroacetic acid, BDCAA: bromodichloroacetic acid, DBCAA: dibromochloroacetic acid.

b Excluding 3.03% of samples with HAA9 < MRL.

Drinking water monitoring data sets like UCMR4 are not an independent, random sample, and the modeled effects of utility characteristics (e.g., treatment type) on water quality outcomes should not be interpreted as causation. For this policy analysis, the purpose of modeling HAA levels as a function of utility characteristics is to understand which types of PWS may be more affected by a change in regulation, and how differences between the UCMR4 data sample and the full population of U.S. PWS may bias the results.

Utility characteristics were modeled as categorical effects in multilevel regression models as discussed in Text S1. Briefly, significant (*p* < 0.01) differences in levels of each HAA group were observed between some or all categories of source water, residual disinfectant and season (Table S2). PWS size was not associated with statistically significant differences in HAA5 or HAA9 levels, but for HAA6Br there was a statistically significant (*p* < 0.05) difference, with small PWS having 7% lower HAA6Br concentrations compared to large PWS. Because only 800 small PWS were required to sample for HAAs under UCMR4, small systems are underrepresented compared to the full US population of PWS. This could be an important limitation for the policy analysis because smaller PWS are associated with higher rates of regulatory violations.^42,43^ However, the present findings suggest that HAA6Br levels may actually be lower in small systems.

Notable differences (>10%) were only observed between certain source waters and residual disinfectant types. The most substantial differences were observed between groundwater and surface water, with mean concentrations that were 71%, 65%, and 39% lower in groundwater than surface water for HAA5, HAA9, and HAA6Br, respectively. These results echo findings by Furst et al. that source water type explained more variance in the relationship between THMs and haloacetonitriles than any other utility characteristic.^18^ Groundwater systems are underrepresented in UCMR4, at 41% of UCMR4 PWS versus ∼80% of all U.S. PWS subject to the D/DBPRs.^16^ As groundwater systems typically have report lower HAA levels than surface water systems,^16^ the percentage of UCMR4 PWS that are implicated by potential limits on HAA9 or HAA6Br is likely an overestimate of the full U.S. population of PWS.

### Scenario 1: Current HAA5 MCL as an Indicator of High HAA6Br Levels

For the HAA5 MCL to be an effective indicator for high brominated HAA levels, there must be a strong probability of co-occurrence of HAA5 MCL exceedances with high HAA6Br levels. Thus, the outcome variable of the models used for the following indicator analysis is the probability that an HAA5 MCL exceedance co-occurs with an equivalently high level of HAA6Br (MCLeq). The analysis was conducted in two phases: First, binary–binary logistic models were used to calculate the probability of an HAA5 MCL exceedance co-occurring with an HAA6Br MCLeq exceedance. Second, logistic regression models were used to evaluate the probability of an HAA5 MCL exceedance co-occurring with *any level* of HAA6Br. As there is no established public health goal level for HAA6Br, a limit was defined to be equivalent to the HAA5 MCL on the basis of percentile (Model A) or the percentage of PWS that are implicated by at least one exceedance (Model B). The HAA5 MCL (60 μg/L) is the ∼98.4th percentile HAA5 LRAA concentration and implicates 4.5% of PWS in UCMR4. The HAA6Br MCLeq is 27.9 μg/L on the basis of percentile LRAA concentration, or 25.4 μg/L on the basis of PWS implicated. Multiple MCLeq definitions were tested to evaluate the model sensitivity to small changes in limit levels.

The probability of the HAA5 MCL identifying an exceedance of the HAA6Br MCLeq defined on the basis of percentile was 0.10, with a 95% confidence interval (CI) of 0.057–0.17 (Table S5). The probability of the HAA5 MCL identifying an exceedance of the HAA6Br MCLeq defined on the basis of PWS was essentially unchanged at 0.11 (CI: 0.060–0.18). In other words, there is only a ∼1 in 10 chance of an HAA5 MCL exceedance co-occurring with equivalently high HAA6Br levels across UCMR4 the sample set. This is an unambiguously poor success rate for a regulatory indicator. However, it is possible that some high HAA6Br and HAA5 levels occur within the same PWS, but at different times. For example, if a source water has consistently high TOC but experiences seasonal fluctuations in bromide (e.g., due to industrial emissions), HAA levels could be high throughout the year with significant fluctuation in bromine substitution. To determine if this was the case, the percentage of PWS that reported at least one exceedance of the HAA5 MCL and the HAA6Br MCLeq at any time was calculated. Only 14–16% of all PWS with an HAA6Br MCLeq exceedance were implicated by at least one exceedance of the HAA5 MCL. Thus, most of the water systems that have high HAA5 do not also have high HAA6Br.

**Figure 1:**
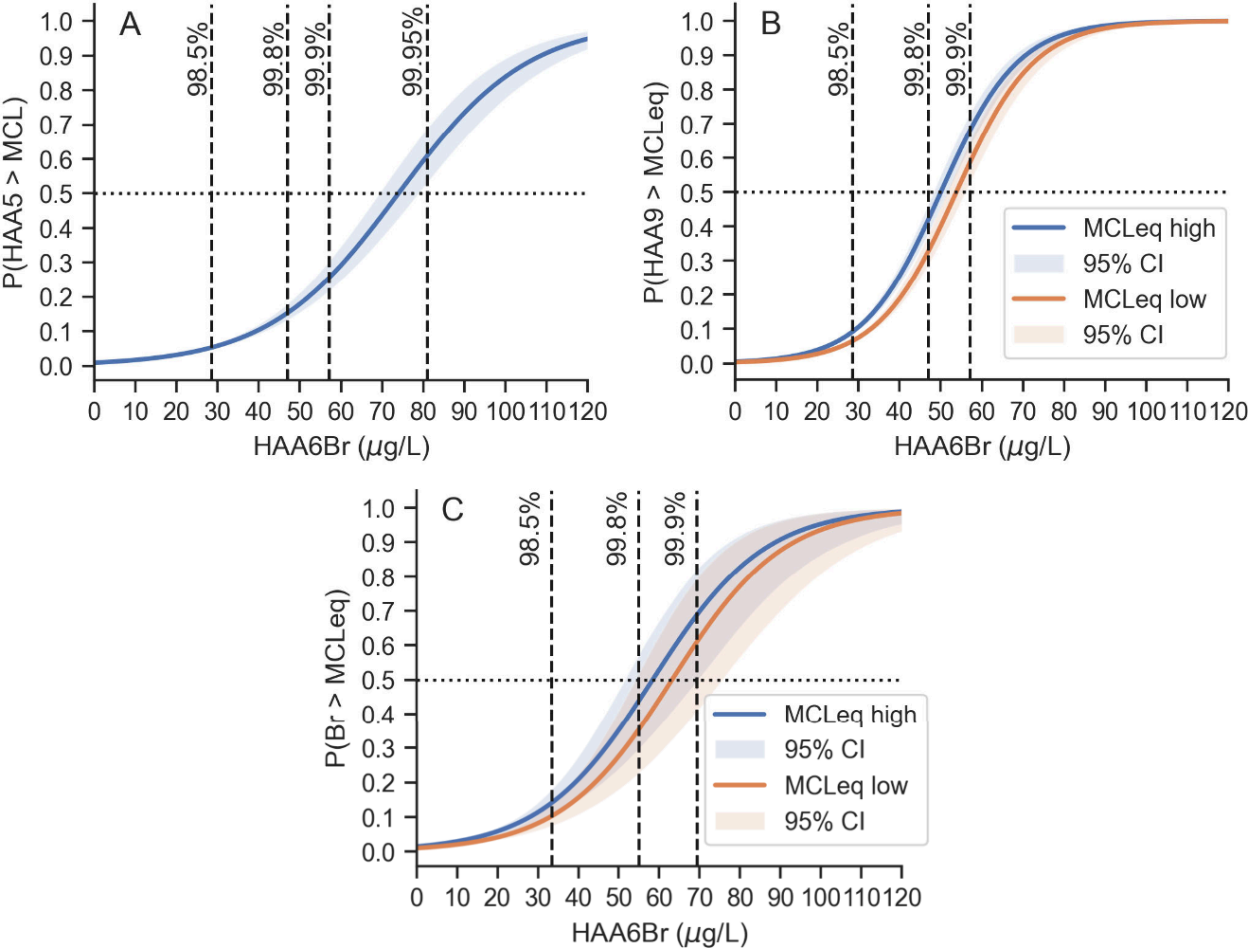
Probability of an exceedance of the A) HAA5 MCL, B) HAA9 MCLeqs, or C) bromide MCLeqs co-occurring with any HAA6Br LRAA concentration. Shaded bands show 95% confidence intervals. The black horizontal dotted line marks the 50% probability line, and the vertical dashed lines demarcate high percentile HAA6Br concentrations discussed in the text. In panel C, HAA6Br concentrations are the maximum LRAA coinciding with each source water bromide measurement. The *x*-axes were truncated from maxima of 165 μg/L (A and B) and 146 μg/L (C) for visibility.

The probability of an HAA5 MCL exceedance co-occurring *with any level* of HAA6Br was modeled with logistic regression (Figure 1A). In the absence of a health advisory level for HAA6Br, this provides an estimate of the indicator efficacy at any level that may be of interest in the future. The result shows that the HAA5 MCL has a less than 1 in 5 probability of co-occurring with the 99.8th percentile HAA6Br level (47 μg/L), and 1 in 4 probability of co-occurring with the 99.9th percentile HAA6Br level (57 μg/L). These are exceptionally high HAA6Br levels, respectively 5.7 and 7.2 standard deviations above the mean of 7.2 μg/L in UCMR4. Only at 99.95 percentile HAA6Br levels (73 μg/L, 10.5 standard deviations above the mean) did the HAA5 MCL improve beyond a 50/50 chance of co-occurrence.

The poor performance of HAA5 as an indicator for HAA6Br is consistent with observations that the majority of HAA6Br consists of unregulated HAAs on a mass basis (Table 1), and that the unregulated brominated HAAs dominate at moderate and high bromide levels.^19,20^ As the HAA5 MCL does not identify the majority of the highest HAA6Br levels, regulatory revisions are needed to effectively limit HAA6Br levels in U.S. drinking water.

### Scenario 2: HAA9 MCL as an Indicator of High HAA6Br Levels

Setting an MCL for HAA9 is considered the likely regulatory route for addressing unregulated brominated HAAs in the US.^23^ Samson and Seidel (2022) identified an HAA9 MCL equivalent (MCLeq) concentration of 72–77 μg/L which would implicate the same number of PWS as the HAA5 MCL. The minimum and maximum of this range are the 98.5th and 98.9th percentile HAA9 LRAA levels in UCMR4, respectively, and would implicate 4.4% or 3.3% of PWS by at least one exceedance (Table S5). The probability of co-occurrence of these HAA9 MCLeq indicators with high HAA6Br levels was evaluated with a set of three logistic models (Table S5). As in Scenario 1, the HAA6Br limits were defined to be equivalent to the HAA9 MCLeqs by percentile (Models A and C) or by percentage of PWS implicated (Model B, ∼3.4% of UCMR4 PWS). Across these models, the mean probability of an HAA9 MCLeq exceedance identifying an HAA6Br MCLeq exceedance ranged from 0.22–0.25 (CIs approximately ±0.10). In practical terms, the success rate of the HAA9 MCLeqs is about 1 in 4, regardless of small changes in MCLeq levels. Of the PWS that reported an HAA6Br MCLeq exceedance, only 24–34% were implicated by at least one HAA9 MCLeq exceedance. Thus, an HAA9 regulation at the MCLeq levels could be twice as effective as the HAA5 MCL, but would still not address the majority of excessive HAA6Br levels. Furthermore, due to the exceptionally high false positive rate (75–78%), the majority of implicated PWS would not have high HAA6Br.

The probability of an HAA9 MCLeq exceedance co-occurring with *any level* of HAA6Br was modeled with logistic regression (Figure 1B). The HAA9 MCLeqs only achieve a 50/50 chance of co-occurrence with HAA6Br levels between the 99.8th (47 μg/L) and 99.9th (57 μg/L) percentiles. Though this performance is better than for HAA5, whether it is good enough depends on what HAA6Br levels are of concern for health. As a thought experiment, if the bulk carcinogenicity of HAA6Br is ∼3 times more potent than HAA5 as estimated by Evans et al.,^15^ we could extrapolate that a health-based limit for HAA6Br should be 20 μg/L (∼1/3rd of the HAA5 MCL). At this limit, HAA9 MCLeqs would identify less than 10% of HAA6Br exceedances; even if it were doubled to 40 μg/L, the HAA9 MCLeqs would only identify ∼20% of such exceedances. This thought experiment and the results of Evans et al. follow EPA’s framework of regulating DBP classes rather than individual contaminants. However, the relationship between concentration and toxicity of a class-based indicator could vary substantially with levels of the individual compounds.

To evaluate whether a lower HAA9 limit would be more effective, a fourth model (D) was defined with an HAA9 limit of 60 μg/L, and HAA6Br held at the same limit as in Model B and C (Table S5). Lowering the HAA9 limit resulted in a decreased probability of identifying HAA6Br MCLeq exceedances (0.16, CI: 0.12–0.21). Though a greater portion of the high HAA6Br cases were correctly identified (37% true positive rate), the false positive rate increased to 84%. The low HAA9 limit identified 50% of UCMR4 PWS with high HAA6Br, but at the cost of implicating 8.8% of all UCMR4 PWS, ∼80% of which did not exceed the HAA6Br MCLeq. Thus, limiting HAA9 would likely be an ineffective and inefficient way to address high brominated HAA levels.

The poor performance of HAA9 as an indicator for HAA6Br may be explained by the fact that the highest HAA9 levels are mainly driven by HAA5. At both HAA9 MCLeq levels, the median contribution of HAA5 to HAA9 is 86%. More specifically, these high HAA9 levels are driven by the chlorine-only species (Cl-HAAs), which were calculated by subtracting HAA6Br from HAA9. The median contribution of Cl-HAAs to the HAA9 mass at the MCLeq levels is 86% for both HAA9 MCLeqs. This suggests that an HAA9 regulatory limit is a better indicator for high Cl-HAAs than for brominated HAAs.

### Scenario 3: Regulatory Limit on Bromide as an Indicator of High HAA6Br Levels

Bromide may be a more effective indicator for high HAA6Br because of the causal relationship between bromide and brominated HAA formation. The relationship between UCMR4 source water bromide levels and each HAA group was evaluated with multilevel regression models to account for repeated measures by PWS (Text S2, Table S4). Additionally, rank correlation coefficients (*r*_*s*_) were calculated between bromide concentrations and bromide levels with each HAA group (Text S2).

**Figure 2:**
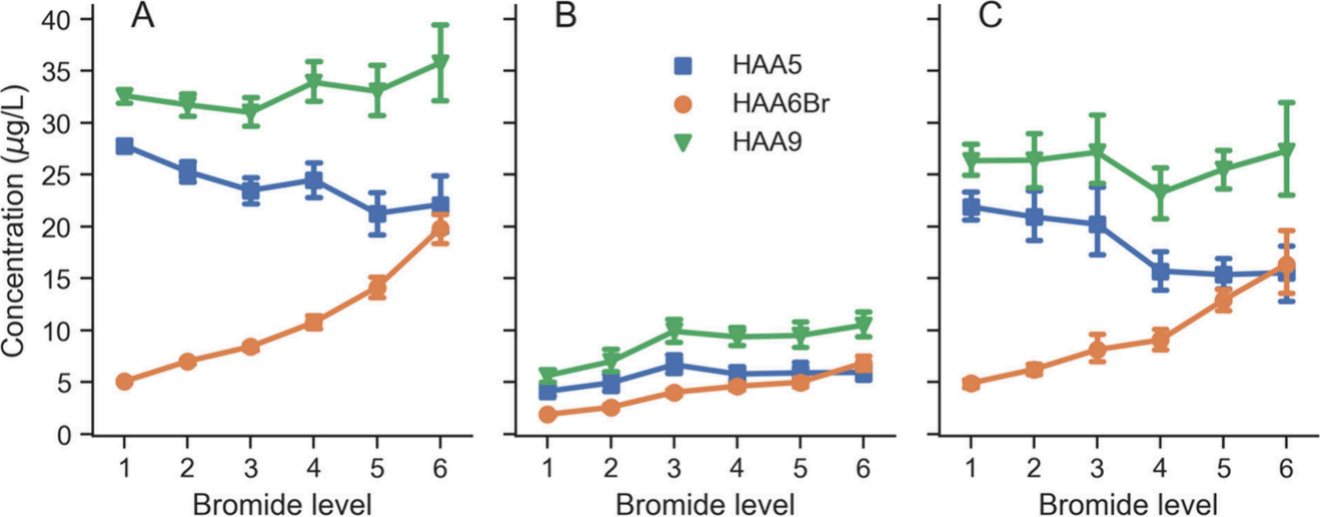
Mean LRAA concentrations of HAA5, HAA6Br, and HAA9 by bromide level for PWS utilizing A) surface water, B) groundwater, or C) mixed surface and groundwater. Bromide bin maxima are 20.0, 30.0, 42.6, 67.0, 124, and 992 μg/L. Error bars represent standard error of the mean. Groundwater under the influence of surface water was excluded due to small sample numbers.

Across UCMR4 water systems, HAA6Br levels reliably increased with bromide level (*r*_*s*_ of 0.27) across source water types while HAA5 and HAA9 levels did not (*r*_*s*_ of −0.30 and −0.19, respectively). All rank correlation *p*-values are less than 0.001 (Table S6). These opposing trends are most striking for PWS utilizing surface water sources, in which mean HAA6Br concentrations increase substantially with each bromide level while mean HAA5 concentrations generally decline (Figure 2A). The net result is that HAA9 concentrations remain relatively flat across bromide levels. In groundwater sources, HAA9 levels initially increase with bromide, up to level 3 (∼43 μg/L) (Figure 2B). This may be explained by HAA6Br comprising a larger portion of HAA9 in groundwater (66%) compared to surface water (28%) on a mass basis among UCMR4 samples. Groundwaters typically have higher bromide/TOC ratios compared to other source waters (Figure S1), which can result in higher BIFs.^26^ Mixed surface and groundwater exhibited intermediate trends, as expected (Figure 2C).

The positive relationship between bromide and HAA6Br levels suggests that bromide could be a better indicator for HAA6Br than HAA9. To test this hypothesis, bromide limits were selected to be equivalent to the HAA9 MCLeqs on the basis of PWS percentage affected (3.3% and 4.4% of UCMR PWS). These bromide MCLeqs are 374 μg/L and 314 μg/L, respectively, and are the 97.8th and 96.9th percentile bromide concentrations in UCMR4. The probability of the bromide MCLeqs co-occurring with equivalently high HAA6Br levels was tested in a series of logistic models (Table S5). As for Scenario 2, the HAA6Br benchmarks were set to be equivalent to the bromide MCLeqs by percentile (Models A and C), or by percentage of PWS implicated (Model B). Across these models, the mean probability of a bromide MCLeq exceedance co-occurring with an HAA6Br limit exceedance was 0.23–0.26 (CIs approximately ±0.1). In other words, there is a 1 in 4 chance of a bromide MCLeq exceedance correctly identifying high HAA6Br levels. At best, the bromide MCLeqs identified only 30% of PWS with equivalently high HAA6Br levels. Surprisingly, this performance is no better than HAA9.

Subsequently, a much lower bromide limit of 200 μg/L (93.9th percentile) was tested. This limit would implicate 8.8% of UCMR4 PWS and is equivalent to the 60 μg/L HAA9 limit tested in Scenario 2 (Model D). At this low bromide limit, the probability of co-occurrence with the HAA6Br MCLeq is just 0.19 (CI: 0.14–0.25), though the true positive rate (42%) is the highest of any model tested (Table S5). Only ∼50% of the PWS that exceeded the HAA6Br MCLeq were successfully identified. The majority of implicated PWS (80%) did not report any exceedance of the HAA6Br MCLeq The poor performance of the bromide indicator is remarkably similar to that of HAA9. Regardless of the indicator, setting a lower limit will capture more PWS with high HAA6Br levels, but will needlessly penalize the majority of implicated PWS.

As for Scenarios 1 and 2, the probability of the bromide MCLeqs co-occurring *with any level* of HAA6Br was evaluated by logistic regression (Figure 1C). As for HAA9, the bromide MCLeqs achieve a 50/50 chance of co-occurrence with HAA6Br levels between the 99.8th and 99.9th percentiles. However, the 95% CI bands for the bromide model are notably wider than for the HAA9 or HAA5 models, reflecting the more limited sample size for the bromide analysis. Ultimately, the unintuitive finding that bromide performs just as poorly as HAA9 as an indicator of high HAA6Br levels underscores the value of this quantitative approach to evaluating indicator efficacy.

### Scenario 4: Impact of an HAA6Br Limit on U.S. Water Systems

**Figure 3:**
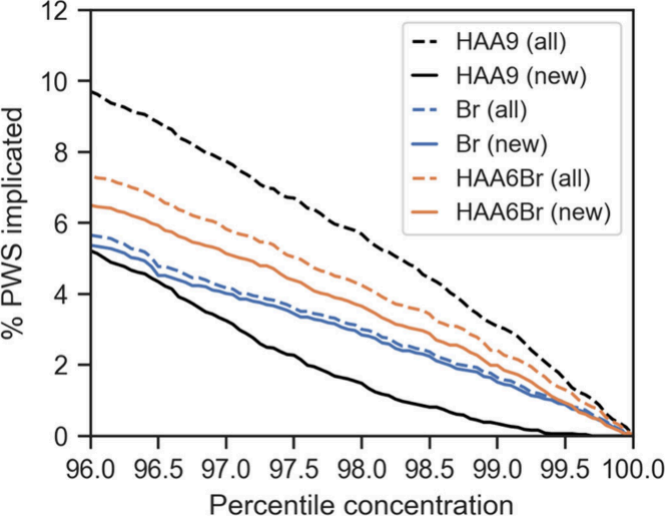
Percentage of PWS implicated by at least one result in excess of a percentile-based limit on HAA9 or HAA6Br LRAA concentrations, or source water bromide concentrations. Dashed lines include all PWS, while solid lines exclude any PWS that also reported at least one HAA5 LRAA result above the MCL.

A regulatory limit for either HAA9 or bromide must be set unreasonably low in order to identify just ∼50% of PWS with high HAA6Br, which would needlessly implicate hundreds of PWS without high HAA6Br. Thus, an HAA6Br limit would be the most direct approach to address excessive brominated HAA levels in drinking water. However, an HAA6Br limit could newly implicate more U.S. PWS than an equivalent HAA9 or bromide limit (Figure 3). Most PWS that might be affected by an HAA9 limit are already implicated by the HAA5 MCL. For example, at HAA9 MCLeq levels (98.5–98.9th percentile), 82–88% of the implicated PWS are already implicated by the HAA5 MCL. A bromide limit would newly affect more PWS than HAA9, but with little benefit for addressing high HAA6Br levels. Most PWS that may be affected by an HAA6Br limit were not already implicated by the HAA5 MCL. For example, an HAA6Br limit at the 98.5th percentile level implicated 3.4% of UCMR4 PWS, ∼85% of which did not have an HAA5 MCL exceedance. These PWS were investigated to understand the potential impacts of an HAA6Br limit on U.S. water systems.

Of the 142 UCMR4 PWS that could be newly implicated by an HAA6Br limit at the 98.fifth percentile level, 91% are large, compared to 85% of UCMR4 PWS. These PWS are distributed across 22 states and territories (Figure S4), but a disproportionate number (47%) are located in Region 6, compared to just 14% of all UCMR4 systems. Most of these are in Texas (37% of implicated PWS versus 8.7% of UCMR4). Region 9 has the next highest number of implicated PWS, with 17% of implicated PWS versus 12% of UCMR4. Most of the implicated Region 9 PWS are in California, with 14% implicated (versus 9.3% of UCMR4). Puerto Rico is the most drastically overrepresented state or territory, with 11% of implicated PWS versus 1.4% overall. Of the 67 Puerto Rican PWS in UCMR4, 16 were implicated, suggesting inequities in exposure to brominated HAAs, as well as in the potential financial burden of complying with an HAA6Br limit.

Surface water was used at 54% of implicated PWS, compared to 45% of all UCMR4 PWS. Most of the implicated Texas PWS use surface water (36 out of 53); the median bromide concentration of these systems is 371 μg/L, corroborating previous findings of high bromide levels in some Texas watersheds.^30^ Groundwater PWS comprise only 21% of implicated PWS versus 41% in UCMR4, which is consistent with typically low TOC levels. Louisiana and Florida are tied for most implicated groundwater systems (n = 6); both coastal states have groundwater supplies threatened by seawater intrusion, which is associated with elevated bromide levels.^44^ Implicated PWS using mixed surface and groundwater sources are overrepresented at 25% versus 14% in UCMR4. Of the implicated mixed sources, 40% are in Texas and 34% in California. Mixed sources tend to have moderately high bromide/TOC ratios (Figure S4). Furthermore, some PWS may mix conventional surface or groundwater with unconventional sources possessing high bromide, though these sources were not reported in UCMR4.

Many PWS implicated by high HAA6Br levels reported using treatment techniques that can help minimize DBP levels. Almost half (48%) used alternatives to chlorine for primary or secondary disinfection, compared to 28% of all UCMR4 PWS. Chloramine was used at 44% of implicated PWS, followed by chlorine dioxide at 15%. Compared to chlorine, chloramination can lower HAA formation by ∼70–90%,^45^ and sequential chlorine-chloramine by ∼20–50%.^46^ Chlorine dioxide forms minimal levels of dihalogenated HAAs.^45^ Almost a third (32%) of implicated PWS using alternative disinfectants also reported using chlorine, which may have contributed to the high HAA6Br levels. EPA-designated DBP precursor removal BATs are enhanced coagulation or softening with GAC, and nanofiltration.^24^ PWS were classified as using a BAT if they reported using GAC or any membrane process, as UCMR4 did not specify enhanced coagulation or membrane type. BATs (membranes and/or GAC) were implemented at 15% of implicated PWS at the time of UCMR4. For the implicated PWS that have not implemented alternative disinfectants and/or precursor removal BATs, doing so may help comply with an HAA6Br limit.

This study examines potential revisions to the HAA rule, and assumes that no concurrent regulatory revisions would occur. The main results are notably robust, with large effect sizes and *p*-values significantly less than 0.001, indicating that the overall conclusions may be resilient to changes among a subset of PWS over time. As always with DBP regulations, there is concern that new limits will motivate utilities to compromise disinfection efficacy, e.g., by cutting chlorine doses. If this were to occur, the potential public health benefits could be negated by increased microbial risk. Evaluating that potential is beyond the scope of this study. A full cost-benefit analysis, including the economic impact to utilities, is also beyond the scope of this study, and would be commissioned by EPA if these scenarios are considered.

### Implications

The Stage 1 and Stage 2 D/DBPRs broadly led to lower DBP levels, and likely lower exposure risk, in many water systems.^22^ Now, regulators are looking to target more specific classes that were not directly addressed by these rules and may pose outsized risks, such as brominated HAAs. Currently, there is no standard for evaluating proposed regulatory indicators. The indicator analysis approach proposed in this study directly aligns with the drinking water regulatory framework used internationally, such that the outcome metrics can objectively inform policy development. This approach uses statistical models which are commonly used in the relevant fields of environmental epidemiology and risk assessment, and can be implemented in any data analysis software.

A general principle can be derived from this study, which is the trade-off between precision and comprehensiveness in setting the limit of a regulatory indicator. Lowering the limit of a regulatory indicator increases the sample size of positive hits, thereby increasing the numbers of both true positives and false positives. For drinking water regulations, false positives represent water systems that may be penalized despite not being a source of high exposure risk. This “collateral damage” may be acceptable if the rate of true positives (i.e., water systems that do have elevated levels of the target) is high enough, and the target itself would be too difficult to regulate directly.

Of the regulatory scenarios EPA is considering to address brominated HAAs, limiting HAA6Br would likely be the most effective. However, HAA6Br is really an indicator for the cumulative risk of exposure to brominated HAAs, and could be ineffective if their cumulative toxicity is not closely linked to concentration. Peterson et al. concluded that HAA6Br concentrations are not strongly correlated with cumulative toxicity because it is controlled by the lowest concentration species, bromoacetic acid.^36^ However, this conclusion relied on *in vitro* cytotoxicity values that may substantially overestimate the toxic potency of bromoacetic acid. California’s Public Health Goal level for dibromoacetic acid (0.03 μg/L) is almost 4 orders of magnitude lower than for bromoacetic acid (25 μg/L).^47^ This substantial discrepancy between a single *in vitro* toxicity assay and California’s Public Health Goals, developed through expert review of all *in vivo* and *in vitro* data, underscores the importance of establishing human health advisory levels for the other brominated HAAs.

Until health-based advisory levels are established for all brominated HAA species, it is not possible to determine whether HAA6Br is an effective indicator of risk. Indeed, evaluating risk is beyond the scope of the present study. Once health advisory levels are available for all brominated HAAs, future work should investigate whether HAA6Br is a sufficient indicator for cumulative risk. If HAA6Br is found to be insufficient, an alternative class-based regulatory approach that could be evaluated is the hazard index, in which a limit is applied to the sum of the concentration of each species weighted by its health-based advisory level. This approach is recommended by the World Health Organization for THMs and was recently implemented by EPA for four per- and polyfluoroalkyl substances (PFAS).^48^

Further research is needed to determine whether regulating HAA6Br could have indirect public health benefits through addressing other toxic brominated DBPs, as noncompliant water systems switch water sources or upgrade treatment processes. Similarly, a bromide limit could have broader benefits by reducing other brominated DBPs, despite its poor performance as an indicator for HAA6Br. Furthermore, bromide may serve as a better indicator for other brominated DBP classes, such as haloacetonitriles, which tend to have a higher BIF than HAAs and THMs and thus may be more sensitive to bromide level.^27,49^ Finally, although EPA is not considering revisions to the THM rule, future work could evaluate whether regulating brominated THMs either individually or as a class would indirectly limit brominated HAAs, and vice versa. Previous work suggested that regulating brominated THMs separately from chloroform may be necessary to address their greater carcinogenicity.^31,50^ Any new regulations targeting brominated DBPs would need to be accompanied by financial and technical support for small and underserved water systems, particularly if bromide removal is required.

In principle, the analytical approach presented in this work can be used to generate direct answers to all these research questions regarding the efficacy of current and potential regulatory indicators.

## Supporting Information Available

Additional methods detail and sensitivity analyses; additional tables and figures providing detailed data summaries and modeling results (PDF)
