# Supplementary material for: Quantitative Evaluation of Regulatory Indicators for Brominated Haloacetic Acids in Drinking Water
Source: Environ Sci Technol. 2025 Feb 25;59(9):4245–54. doi: 10.1021/acs.est.4c10202 (PMC11912320; doi:10.1021/acs.est.4c10202)
Supplement: Supplementary file 1 — es4c10202_si_001.pdf [file es4c10202_si_001.pdf]

Supporting Information:

Quantitative evaluation of regulatory indicators for brominated haloacetic acids in drinking water

Kirin Emlet Furst[1,2*]

[1] Sid and Reva Dewberry Department of Civil, Environmental & Infrastructure Engineering, George Mason University, Fairfax, VA, 22030, United States

[2] Occoquan Watershed Monitoring Laboratory, The Charles E. Via, Jr. Department of Civil and Environmental Engineering, Virginia Tech, Manassas, VA, 22152, United States

*kfurst@vt.edu

Summary: 20 pages, 3 texts, 6 tables, 4 figures.

TABLE OF CONTENTS

TEXT S1: RELATIONSHIP BETWEEN PWS CHARACTERISTICS AND HAA LEVELS ... 3

TEXT S2: BROMIDE ANALYSIS ... 4

2.1. Bromide data screening: High bromide levels and membrane treatment ... 4

2.2. Bromide multilevel regression trend analysis ... 6

TEXT S3: VALIDATION OF LOGISTIC REGRESSION MODELS ... 6

3.1. Independence of observations ... 6

3.2. Influence of outliers ... 7

3.3. Assumption of linearity between independent variable and log-odds ... 8

3.4 Sensitivity analysis on left-censored values ... 8

SUPPLEMENTARY TABLES: S1–S6 ... 9

SUPPLEMENTARY FIGURES: S1–S4 ... 17

REFERENCES ... 20

Text S1: Relationship between PWS characteristics and HAA levels

The final dataset consisted of 63,427 complete records representing 4,924 U.S. PWS, which reported between 1 and 88 records each. The number of PWS represented in each category (facility size, source type) and the mean, median and maximum concentrations of each HAA group are provided in Table S1. Some types of PWS (e.g., large surface water facilities) are overrepresented in UCMR4 relative to the full population of US water systems. The potential implications of this for the policy analysis are discussed below. The magnitude and statistical significance of differences in mean HAA levels between PWS categories were evaluated using multilevel regression models (MLM) to account for clustering (repeated measures) of data by PWS (Table S2, A–C). These models are intercept-only with categorical predictor variables (size, source water type, and residual disinfectant type), such that the outcome variables are mean levels of HAA5, HAA9, and HAA6Br for each category, as described in more detail in Furst et al. (2021) (Furst et al., 2021). Differences in mean HAA levels as a function of categorical variables are discussed below. All concentrations discussed are LRAAs.

Source water type: The most substantial differences were observed between groundwater and surface water PWS, with mean concentrations that were 70.5%, 65%, and 39% lower in groundwater than surface water for HAA5, HAA9, and HAA6Br, respectively. For HAA5 and HAA9, both groundwater under the influence (GU) and mixed surface and groundwater sources (MX) featured concentrations significantly lower than surface water ($p<0.05$) and higher than groundwater. However, HAA6Br levels in GU sources were not statistically different from surface water, and were 8.0% higher in MX compared to surface water ($p<0.05$).

Size: PWS size was not associated with statistically significant differences in HAA5 or HAA9 levels, but for HAA6Br there was a statistically significant ($p<0.05$) difference, with small PWS having 7% lower HAA6Br concentrations compared to large PWS. Because only a representative sample of 800 small PWS (defined for UCMR4 as those serving <10,000) was required to sample for HAAs under UCMR4, small systems are underrepresented compared to the full US population of PWS. This could be an important limitation for the policy analysis because smaller PWS have been associated with higher rates of regulatory violations (Allaire et al., 2018; Statman-Weil et al., 2020). The present findings suggest that HAA6Br levels may actually be lower in small systems, though it should be noted that the results for small water systems are not as robust as for large systems due to smaller sample sizes and fewer PWS represented (Table S1).

Season: Reported sampling dates were converted to seasons as follows: Winter, December–February; Spring, March–May; Summer, June–August; Fall, September–November. The difference between winter and each of the other three seasons was statistically significant ($p<0.05$) for all three HAA groups, with the lowest mean HAA concentrations in winter and highest in summer. In terms of practical significance however, the differences were relatively modest, with the least difference for HAA6Br (4.3% less) and greatest difference for HAA5 (8.5% less) in winter compared to summer for the base case of large surface water systems using residual chlorine.

Residual disinfectant: PWS reported disinfectant types and treatment processes for each sample by selecting all that applied from a set of fixed choices provided by the EPA. This resulted in 240 unique disinfectant sequences and 802 unique treatment train sequences, which was too complex to enable quantitative modeling. Therefore, the only treatment category used for quantitative

modeling was residual disinfectant type, as it is a significant factor in HAA formation, was readily interpretable from UCMR4 data, and offered sufficient sample size numbers across the four categories (chlorine, chloramine, chlorine dioxide, and "none"). Chlorine residual use (n=38,640) was indicated by the reagent type and manner of generation: CL2 (liquid), CLGA (gaseous), or CLOF (offsite generated hypochlorite, stored in liquid form). Similarly, chloramine residual (n=18,518) was indicated by the chemical reagent type and manner of addition: CLM (chloramine formed from addition of chlorine and ammonia), CAGC (chloramine formed with gaseous chlorine), CAOF (chloramine formed with offsite hypochlorite), CAON (chloramine formed with onsite hypochlorite), or CAC (mixing of chlorinated and chloraminated water). Chlorine dioxide residual (n=290) was indicated as CLO2. Since all PWS that reported HAAs for UCMR4 are subject to the D/DBPRs, the fourth category, "None" (n=5,979), may represent consecutive systems that purchase treated water already carrying a disinfectant residual. Of these "None" records, about 90% (n=4,999) did not report any disinfectant used in the treatment train. Only ~10% (n=602) reported a residual disinfectant was used in the treatment train (chlorine: 422, chloramine: 207, and chlorine dioxide: 39 records). The remainder reported using a disinfectant during treatment that does not leave a disinfectant residual or form HAAs (e.g., ultraviolet light).

For all HAA groups, the mean concentrations were ~10–15% lower in chloraminating systems than in chlorinating systems. The differences between chlorine and chlorine dioxide were not statistically significant for any HAA group, perhaps due to the small sample size for chlorine dioxide (n=290). The differences between chlorine and "none" were statistically significant ($p<0.05$), but the effect size was practically insignificant (<5% difference in concentrations). Overall, these modest differences between residual disinfectants echo findings by Furst et al. (2021) that disinfectant type explained substantially less variance than source water type in the relationship between THMs and haloacetonitriles. Surface waters typically have higher levels of DBP precursors than groundwaters, and therefore higher DBP levels, even with chloramination. Furthermore, the choice of disinfectant type and treatment train is highly dependent on the source water type. Source water may act as a proxy for these and other aspects of the treatment process that cannot be directly evaluated in these models.

Text S2: Bromide analysis

2.1. Bromide data screening: High bromide levels and membrane treatment

Any source water bromide removed prior to disinfection is not available to form HAAs. The main current technology that can reliably achieve high bromide removal (e.g., 2-log) at full-scale is reverse osmosis (RO). Additionally, some high-pressure nanofiltration membranes approach or achieve 2-log bromide removal in real waters (Chowdhury et al., 2022). UCMR4 did not distinguish between RO and other membrane filtration processes. Thus, all systems that reported using membrane filtration ("MFL", 307 PWS, 4,042 records) were excluded for analyses involving bromide. However, many excessively high bromide levels (e.g., >1.0 mg/L) remained.

The source water categories for UCMR4 did not provide the option of reporting unconventional sources like seawater or brackish water, which can have excessive bromide levels. Drinking water bromide levels are not regulated in the US, but excessively high bromide is often found in high salinity waters that require desalination by techniques that will also remove bromide (namely, reverse osmosis, RO) prior to distribution as drinking water. The highest bromide concentration reported in UCMR4 is 72.5 mg/L, which is towards the upper end of the bromide

concentration range of seawater (60–70 mg/L) (Ged and Boyer, 2014). This bromide level was reported by a desalination facility in St. Thomas (Virgin Islands) which uses RO. Many desalination systems blend seawater with other source waters, or utilize brackish water with lower salinity. Some of these PWS may not use RO, depending on salinity of the blended water, or on size and regulatory category.

For screening purposes, 1.0 mg/L bromide was selected as the "high bromide" benchmark, and PWS with mean bromide in excess of this benchmark were investigated (top 0.7%, or 99.3 percentile concentrations). Sixty PWS reported mean bromide in excess of the 1 mg/L benchmark. Of these, 34 reported using membrane filtration (MFL) during at least one sampling event and were excluded as mentioned previously. The 29 PWS that did not report using MFL during at least one sampling event had a somewhat higher mean bromide concentration (5.4 mg/L) than PWS that did report using MFL (4.1 mg/L), and the range of mean bromide concentrations for these systems was 1.0–46 mg/L. Eight of the systems are classified as small. Nine large systems in FL treated groundwater with mean bromide ranging from 1.2–7.0 mg/L using a variety of treatment train combinations, including air stripping and softening. Two large systems in CA had the highest mean bromide levels (38 and 46 mg/L). Neither system reported using MFL, but public information indicates that they do use RO for desalination (City of Santa Barbara, n.d.; Monterey Water, 2022). Several other systems were verified to use RO, despite not selecting MFL in UCMR4. One large system in VA treats brackish groundwater with mean bromide concentration of 4.5 mg/L, and selected "OTH" in UCMR4 despite public information indicating the system uses RO (City of Chesapeake, n.d.). These findings called into question the validity of treatment train data reporting in UCMR4. Thus, systems with bromide in excess of 1 mg/L were excluded from the trend analyses involving bromide. The final bromide dataset excluding bromide >1 mg/L consisted of 8,647 records from 3,164 PWS.

A sensitivity analysis was done to examine the effect of excluding >1 mg/L bromide concentrations ("outliers") on the indicator analysis outcomes (Table S5, Scenario 3 Models A and B). 1 mg/L is the 99.98th percentile concentration, such that excluding concentrations above 1 mg/L excludes the highest 0.02% bromide concentrations. While this is a small percentage of data excluded, it does change the percentile concentration distribution. For Model A, the 98.5th percentile bromide limit excluding outliers is 436 μg/L; for the sensitivity analysis without excluding outliers, the 98.5th percentile bromide limit is 543 μg/L. With outliers excluded, the mean probability of co-occurrence with 98.5th percentile HAA6Br is 0.22; for the sensitivity analysis without exclusions, the mean probability of co-occurrence is much lower at 0.14. This would seem to be a notable difference, but in both cases the 95% confidence intervals are quite wide, such that there is substantial overlap. Models B and C test a lower bromide limit (95th percentile), and the differences between the models with or without outliers are negligible (within 1%). This may be explained by a smaller discrepancy at the 95th percentile bromide concentrations with outliers (254 μg/L) vs. without outliers (228 μg/L).

Overall, there is a relatively minor effect of excluding bromide outliers on the probability of co-occurrence between high levels of bromide and HAA6Br, though the effect of exclusion is greater at higher bromide limits. The effect of outlier exclusion at higher bromide limits is an increase in the probability of co-occurrence with high HAA6Br levels, which is consistent with the assumption that many of the water systems with >1 mg/L source water bromide do not actually have 1 mg/L bromide when the water is disinfected, due to RO treatment, dilution, or other reasons.

2.2. Bromide multilevel regression trend analysis

The relationship between bromide, HAA group and source water type may be influenced by repeated measures by PWS and dependencies between these and other variables. For example, since PWS were not required to report quarterly results over 4 seasons, there may be an asymmetrical number of samples for one season vs. others, which could systematically bias the analysis if there are seasonal effects in any of the indicator or outcome variables. To investigate these systematic effects, multilevel regression models with random intercepts were developed for each HAA group to evaluate the relationship with bromide level while accounting for the effect of clustering by PWS and differences between source water type, disinfectant, and season (Table S4). Note that the coefficients in the MLM output tables represent the mean effect of each bromide level by comparison to the reference case ("Intercept"), which is level 1 bromide, chlorinating surface water systems in winter.

The results show that for HAA6Br, each increase in bromide level is associated with a highly statistically significant ($p<0.001$) increase in HAA6Br level (Table S4C). Level 6 bromide concentrations (>124 μg/L) are associated with mean HAA6Br levels that are ~63% higher than Level 1 concentrations (<20 μg/L). By contrast, the only statistically significant difference ($p<0.05$) in HAA5 levels was a decline of ~1.1–1.2 μg/L between bromide level 1 and levels 5 and 6 (Table S4A). For HAA9, there were statistically significant ($p<0.05$) yet minor increases in concentration between bromide Level 1 and Levels 3 (3%), 4 (3.2%), and 6 (5.6%). These model results support the trends observed in Figure 2 and the interpretation thereof.

To investigate the overall relationship between each HAA group and bromide across source waters with a more common statistical approach, the rank correlation coefficients (Spearman's rho, r_s) were calculated on the same dataset used in the multilevel model. The r_s were calculated with bromide on a concentration basis as well as binned into the six levels, to understand how this might affect the rank correlation significance. The findings demonstrate that across all source waters, bromide has a significant positive correlation with HAA6Br and significant negative correlations with HAA9 and particularly HAA5 (Table S6). All correlations were highly significant (p-values significantly less than 0.001). The rank correlations are not meaningfully affected by binning the bromide levels. For example, the r_s between bromide concentrations or bromide levels with HAA6Br are 0.25 and 0.27, respectively, both with very low p-values of 6.3×10^{-96} and 1.6×10^{-74} due to the large sample size.

Text S3: Validation of logistic regression models

The assumptions of logistic regression differ from those of linear regression. Unlike linear regression, logistic regression does not require a linear relationship between independent and dependent variables, normal distribution of the residuals, or homoscedasticity. The following assumptions relevant for the logistic regression models used in this study are discussed and validated here: 1) the observations are independent of each other; 2) the sample size is sufficiently large that "outliers" do not have an outsized effect; 3) the linearity of independent variables and log odds is assumed.

3.1. Independence of observations

The UCMR4 data violate the assumption of the independence of errors because of "repeated measures" (i.e., multiple samples from each PWS) which can result in correlated residuals among

samples from the same PWS. Using the sample data without accounting for this clustering by PWS could underestimate the associated uncertainty (error). The logistic indicator models ("Stage 1") were programmed using the python module statsmodels (Seabold et al., 2010) to account for clustering by PWS in the covariance matrix for calculating the errors, which are reported as 95% confidence intervals. The difference with and without clustering by PWS was examined for Scenario 1 and Scenario 2. For Scenario 1, the probability of co-occurrence without accounting for clustering was estimated as 0.10 (0.084–0.12), and with clustering, was 0.10 (0.057–0.17). For Scenario 2 Model A, the probability of co-occurrence was estimated as 0.23 (0.21–0.26) without accounting for clustering, and with clustering, 0.23 (0.16–0.33). Thus, in both cases, the estimated mean probabilities were essentially unchanged by accounting for clustering, but the 95% confidence interval (CI) bands were wider when accounting for clustering. This is consistent with underestimation of errors when a dependent data sample is treated as independent. Based on this comparison, all logistic models reported in Table S5 incorporate the robust error estimate to account for clustering.

For the logistic regression models with continuous predictor variables (Figure 1), the option to account for clustering in model errors was not available, such that the analysis presented in the main text is on pooled data. To determine whether the pooling of repeated measures may have biased the results, the analysis was repeated for HAA5 and HAA9 with LRAA concentrations averaged by PWS (Figure S2). Completely unpooling the data eliminates the issue of correlated errors, but discards the within-PWS variance between sampling locations and seasons, and underestimates the number of benchmark exceedances. So the purpose of this exercise is to provide the other extreme for comparison. The HAA5 MCL indicator performs somewhat worse on the unpooled data vs. the pooled data, in that it only achieves a 50/50 probability of co-occurring with high HAA6Br concentrations above the 99.95th percentile (Figure S2A) vs. somewhat below the 99.95th percentile (Figure 1A). For HAA9, the completely unpooled regression (Fig. S2B) performs similarly to the pooled regression (Fig. 1B) in that the MCLeqs reach 50/50 probability of co-occurring with high HAA6Br levels between 99.8th and 99.9th percentile. Overall, the conclusion drawn in the main text is unchanged: the HAA5 MCL and HAA9 MCLeq have low probabilities (<50%) of detecting all but the highest (0.2–0.1%) HAA6Br concentrations. The "true probabilities" are assumed to be intermediate to the pooled (Figure 1) and unpooled (Figure S2) results.

3.2. Influence of outliers

This study strives to understand the real variation in levels of different HAA groups across U.S. PWS. For the regulatory context, we are especially concerned with the highest HAA concentrations. In that sense, there are no outliers, and it does not make sense to remove them. However, they could introduce bias into the statistical model due to the highly skewed (right-tailed) data distribution. Thus, a sensitivity analysis was conducted on the indicator models to understand the effect of removing the highest 0.01% and 0.02% HAA6Br concentrations (Table S5). Removing outliers had no practical or statistically significant effect on the probability of the indicator (x=1) coinciding with a high concentration of HAA6Br (y=1). Thus, the conclusions remain the same regardless of inclusion of "outliers": HAA9 is more effective as an indicator of high HAA6Br than HAA5, but depending on the limit selected, only has a 1-in-4 or 1-in-5 chance of identifying a high HAA6Br concentration.

3.3. Assumption of linearity between independent variable and log-odds

This assumption is tested with the Box-Tidwell transformation, in which the independent variable is multiplied by the natural log of each independent variable and tested for significance. The logistic regressions in Table S4 calculate the probability of co-occurrence of an HAA5 MCL or HAA9 MCLeq exceedance with a particular benchmark value for HAA6Br. As these contain binary independent variables, the assumption of linearity is moot. For the logistic regressions plotted in Figures 2 and 3 however, the independent variable is continuous (HAA6Br LRAA concentrations). The Box-Tidwell test was performed for the model with x = HAA6Br (continuous) and y=[HAA9<= or > 72 μg/L] (binary). The result for the transformed independent variable is highly significant ($p<0.001$), which means the test is passed for the assumption of linearity.

3.4 Sensitivity analysis on left-censored values

For each group of HAAs, if all species were below their method reporting limits (MRLs), the sum was reported as 0 μg/L. UCMR4 HAA MRLs were not reported, yet all three HAA groups had minimum (non-zero) values of 0.2 μg/L, so <MRL entries were replaced with 0.2 μg/L. A minor portion of records had <MRL entries, ranging from 5.5% for HAA6Br to 3.0% for HAA9 (Table 1). As LRAA concentrations were used for the analyses, some of these <MRL entries get averaged with >MRL concentrations. The percentage of LRAAs calculated from all <MRL records is as follows: HAA6Br, 3.65%; HAA5, 2.17%; and HAA9, 2.00%. Percentile concentration is important to many of the analyses in this manuscript, and so it is worth noting that these percentages are also the percentiles. The percentiles remain unaffected by the <MRL replacement, because percentile is a rank metric and the replacement does not change the rank order.

The replacement of <MRL (0 μg/L) entries with 0.2 μg/L is not expected to influence any of the analyses or outcomes of this study. Given the distribution of data for each HAA group, with medians and means between 5–25 μg/L, a delta of 0.2 μg/L for the lowest ~5% of concentrations would be of little significance for most contexts. For the particular modeling approaches used in this study, the lowest concentrations have little to no influence on the outcomes.

The core analysis using the logit function (Table S4) is completely unaffected by <MRL replacements. This is because for each HAA group, the concentrations were converted to binary indicator variables by comparison to a benchmark concentration (a limit), with concentrations less than or equal to the benchmark assigned "0" and concentrations greater than the benchmark assigned "1". The model is thus ignorant of the exact value of any concentrations, only whether they are above or below the high benchmark.

For the logistic regression models (Figure 1), HAA6Br is modelled as a continuous variable. However, the lowest ~5% of concentrations have little influence on the fit of a regression model, particularly with such a large dataset and broad distribution of data. This is demonstrated in Figure S3, in which Figures 1A and 1B were replotted with the <MRL = 0.0 μg/L dataset. The results of Figure S3A and S3B are indistinguishable from Figures 1A and 1B, respectively. Thus, the difference of 0.2 μg/L in the lowest concentrations is negligible.

Supplementary Tables: S1–S6

Table S1: Number of UCMR4 PWS* by facility size and source type, with the mean, median, and maximum of mean PWS LRAA concentrations of each HAA group in μg/L.

PWS Size	Source	Group	# PWS	Mean	Median	Maximum
Large	GU	HAA5	53	11.9	8.76	58.7
		HAA6Br	53	8.37	6.71	69.3
		HAA9	53	18.2	14.2	85.3
	GW	HAA5	1551	6.18	2.72	111
		HAA6Br	1551	4.57	2.68	63.7
		HAA9	1551	9.56	4.72	139
	MX	HAA5	688	15.8	12.7	114
		HAA6Br	688	7.58	6.22	68.1
		HAA9	688	21.8	19.4	122
	SW	HAA5	1994	23.0	20.8	188.6
		HAA6Br	1994	7.59	6.38	93.0
		HAA9	1994	29.5	27.5	272
Small	GU	HAA5	9	13.4	5.95	36.9
		HAA6Br	9	5.25	4.67	10.8
		HAA9	9	17.9	10.0	45.1
	GW	HAA5	451	5.07	1.73	255
		HAA6Br	451	3.67	1.37	50.5
		HAA9	451	7.70	2.83	269
	MX	HAA5	24	19.8	19.7	43.7
		HAA6Br	24	6.65	5.40	23.7
		HAA9	24	25.3	25.5	53.7
	SW	HAA5	244	24.6	23.5	129
		HAA6Br	244	7.93	5.82	63.5
		HAA9	244	31.4	28.7	180

Table Key: Surface water (SW), groundwater (GW), groundwater under the influence (GU), and mixed surface and groundwater (MX).

*Note that some PWS changed source type between sampling dates and/or locations, and are thus "double counted" in this table.

Table S2: Multilevel random-intercept regression model to estimate the effect of key categorical variables and clustering by PWS on (A) HAA5, (B) HAA9, and (C) HAA6Br LRAA concentrations; intercepts are the mean concentration of the reference group (large surface water PWS using chlorine in winter); all other coefficients represent the mean difference between that treatment category (T.) and the reference group.

A) HAA5

Model:	MixedLM	Dependent Variable:	HAA5
No. Observations:	63427	Method:	REML
No. Groups:	4924	Scale:	50.2006
Min. group size:	1	Log-Likelihood:	-222478.5134
Max. group size:	88	Converged:	Yes
Mean group size:	12.9		

	Coef.	Std.Err.	z	P>|z|	[0.025	0.975]
Intercept	22.248	0.264	84.169	0.000	21.729	22.766
C(WaterType, Treatment(reference='SW'))[T.GU]	-10.172	1.159	-8.777	0.000	-12.443	-7.900
C(WaterType, Treatment(reference='SW'))[T.GW]	-15.695	0.340	-46.111	0.000	-16.362	-15.028
C(WaterType, Treatment(reference='SW'))[T.MX]	-5.978	0.361	-16.536	0.000	-6.686	-5.269
C(Residual, Treatment(reference='CL2'))[T.CLM]	-2.925	0.275	-10.650	0.000	-3.463	-2.387
C(Residual, Treatment(reference='CL2'))[T.ClO2]	0.235	0.965	0.243	0.808	-1.658	2.127
C(Residual, Treatment(reference='CL2'))[T.None]	-0.805	0.297	-2.705	0.007	-1.388	-0.222
C(Size, Treatment(reference='L'))[T.S]	-0.543	0.513	-1.059	0.289	-1.548	0.462
C(Season, Treatment(reference='WIN'))[T.FALL]	1.205	0.084	14.343	0.000	1.040	1.370
C(Season, Treatment(reference='WIN'))[T.SPR]	0.857	0.084	10.240	0.000	0.693	1.021
C(Season, Treatment(reference='WIN'))[T.SUM]	1.881	0.081	23.234	0.000	1.723	2.040
Group Var	144.937	0.453				

B) HAA9

Model:	MixedLM	Dependent Variable:	HAA9
No. Observations:	63427	Method:	REML
No. Groups:	4924	Scale:	71.3125
Min. group size:	1	Log-Likelihood:	-233652.6995
Max. group size:	88	Converged:	Yes
Mean group size:	12.9		

	Coef.	Std.Err.	z	P>|z|	[0.025	0.975]
Intercept	28.684	0.317	90.452	0.000	28.063	29.306
C(WaterType, Treatment(reference='SW'))[T.GU]	-11.026	1.387	-7.947	0.000	-13.745	-8.306
C(WaterType, Treatment(reference='SW'))[T.GW]	-18.680	0.408	-45.778	0.000	-19.480	-17.881
C(WaterType, Treatment(reference='SW'))[T.MX]	-5.737	0.433	-13.254	0.000	-6.586	-4.889
C(Residual, Treatment(reference='CL2'))[T.CLM]	-3.863	0.329	-11.739	0.000	-4.509	-3.218
C(Residual, Treatment(reference='CL2'))[T.ClO2]	0.178	1.152	0.154	0.877	-2.080	2.435
C(Residual, Treatment(reference='CL2'))[T.None]	-1.124	0.355	-3.164	0.002	-1.821	-0.428
C(Size, Treatment(reference='L'))[T.S]	-0.947	0.616	-1.537	0.124	-2.155	0.261
C(Season, Treatment(reference='WIN'))[T.FALL]	1.366	0.100	13.642	0.000	1.170	1.562
C(Season, Treatment(reference='WIN'))[T.SPR]	1.009	0.100	10.119	0.000	0.814	1.205
C(Season, Treatment(reference='WIN'))[T.SUM]	2.154	0.097	22.313	0.000	1.964	2.343
Group Var	209.720	0.551				

C) HAA6Br

```
==========================================================================================
Model:                    MixedLM          Dependent Variable:          HAA6Br
No. Observations:         63427            Method:                      REML
No. Groups:               4924             Scale:                       8.8673
Min. group size:          1                Log-Likelihood:              -168317.6378
Max. group size:          88               Converged:                   Yes
Mean group size:          12.9
------------------------------------------------------------------------------------------
                                                  Coef.  Std.Err.    z    P>|z| [0.025 0.975]
------------------------------------------------------------------------------------------
Intercept                                          7.596    0.126  60.177 0.000  7.348  7.843
C(WaterType, Treatment(reference='SW'))[T.GU]     -0.533    0.526  -1.012 0.312 -1.564  0.499
C(WaterType, Treatment(reference='SW'))[T.GW]     -2.970    0.160 -18.586 0.000 -3.283 -2.657
C(WaterType, Treatment(reference='SW'))[T.MX]      0.606    0.163   3.726 0.000  0.287  0.925
C(Residual, Treatment(reference='CL2'))[T.CLM]    -1.117    0.123  -9.117 0.000 -1.358 -0.877
C(Residual, Treatment(reference='CL2'))[T.ClO2]   -0.114    0.413  -0.275 0.783 -0.922  0.695
C(Residual, Treatment(reference='CL2'))[T.None]   -0.349    0.130  -2.691 0.007 -0.603 -0.095
C(Size, Treatment(reference='L'))[T.S]            -0.539    0.252  -2.135 0.033 -1.033 -0.044
C(Season, Treatment(reference='WIN'))[T.FALL]      0.209    0.035   5.902 0.000  0.139  0.278
C(Season, Treatment(reference='WIN'))[T.SPR]       0.171    0.035   4.869 0.000  0.102  0.240
C(Season, Treatment(reference='WIN'))[T.SUM]       0.330    0.034   9.708 0.000  0.264  0.397
Group Var                                         36.068    0.264
==========================================================================================
```

Table S2 Key: Source water types (WaterType): Surface water (SW), groundwater (GW), groundwater under the influence (GU), and mixed surface and groundwater (MX). Residual types: chlorine (CL2), chloramine (CLM), chlorine dioxide (CL02), and no residual reported (None). Size: Large (L) and small (S). Seasons: winter (WIN), fall (FALL), spring (SPR), and summer (SUM).

Table S3: Bromide bin sample numbers and distributions by source water type (excluding systems using membrane filtration or reporting samples in excess of 1 mg/L).

			**Bromide concentrations (µg/L)**		
**Source water**	**Level**	***Sample n***	***Minimum***	***Median***	***Maximum***
**All**	1	3464	<20	<20	20
	2	1042	20	25	30
	3	1030	30	36	43
	4	1050	43	53	67
	5	1025	67	88	124
	6	1036	124	201	992
**SW**	1	2140	<20	<20	20
	2	569	20	26	30
	3	463	30	35	43
	4	381	43	52	67
	5	260	68	89	123
	6	283	125	220	955
**GW**	1	787	0	0	20
	2	332	20	26	30
	3	470	30	36	43
	4	550	43	53	67
	5	591	67	88	124
	6	637	124	197	975
**MX**	1	501	0	0	20
	2	121	20	25	30
	3	81	30	37	42
	4	101	43	54	67
	5	152	67	88	122
	6	101	126	194	992
**GU**	1	36	0	0	19
	2	20	20	25	30
	3	16	31	35	43
	4	18	44	58	67
	5	22	68	87	123
	6	15	131	202	720

Table S4: Multilevel random-intercept regression model estimating the effect of key categorical variables and clustering by PWS on the maximum LRAA concentration of (A) HAA5, (B) HAA9, and (C) HAA6Br corresponding to each bromide measurement; intercepts are interpreted as the mean concentration of the reference group (large surface water PWS using chlorine in winter), and coefficients are the mean difference between each treatment category and the reference group.

A) HAA5

Model:	MixedLM	Dependent Variable:	HAA5
No. Observations:	8962	Method:	REML
No. Groups:	3164	Scale:	26.8957
Min. group size:	1	Log-Likelihood:	-31532.9741
Max. group size:	9	Converged:	Yes
Mean group size:	2.8		

	Coef.	Std.Err.	z	P>|z|	[0.025	0.975]
Intercept	24.741	0.380	65.034	0.000	23.995	25.487
Br_bin[T.2]	-0.366	0.276	-1.326	0.185	-0.906	0.175
Br_bin[T.3]	-0.022	0.314	-0.071	0.943	-0.638	0.594
Br_bin[T.4]	-0.481	0.340	-1.414	0.157	-1.148	0.186
Br_bin[T.5]	-1.238	0.380	-3.257	0.001	-1.983	-0.493
Br_bin[T.6]	-1.140	0.444	-2.568	0.010	-2.010	-0.270
C(WaterType, Treatment(reference='SW'))[T.GU]	-10.820	1.614	-6.705	0.000	-13.983	-7.657
C(WaterType, Treatment(reference='SW'))[T.GW]	-18.845	0.435	-43.369	0.000	-19.696	-17.993
C(WaterType, Treatment(reference='SW'))[T.MX]	-5.862	0.582	-10.065	0.000	-7.004	-4.721
C(Residual, Treatment(reference='CL2'))[T.CLM]	-1.789	0.448	-3.991	0.000	-2.668	-0.910
C(Residual, Treatment(reference='CL2'))[T.ClO2]	-0.130	1.344	-0.097	0.923	-2.764	2.504
C(Residual, Treatment(reference='CL2'))[T.None]	-0.306	0.442	-0.692	0.489	-1.174	0.561
C(Season, Treatment(reference='WIN'))[T.FALL]	1.387	0.176	7.898	0.000	1.043	1.731
C(Season, Treatment(reference='WIN'))[T.SPR]	0.705	0.175	4.030	0.000	0.362	1.047
C(Season, Treatment(reference='WIN'))[T.SUM]	1.752	0.161	10.889	0.000	1.437	2.068
Group Var	124.728	0.812				

B) HAA9

Model:	MixedLM	Dependent Variable:	HAA9
No. Observations:	8962	Method:	REML
No. Groups:	3164	Scale:	37.3029
Min. group size:	1	Log-Likelihood:	-33127.8264
Max. group size:	9	Converged:	Yes
Mean group size:	2.8		

	Coef.	Std.Err.	z	P>|z|	[0.025	0.975]
Intercept	30.911	0.462	66.939	0.000	30.006	31.816
Br_bin[T.2]	0.129	0.326	0.395	0.693	-0.511	0.768
Br_bin[T.3]	0.943	0.373	2.531	0.011	0.213	1.674
Br_bin[T.4]	0.996	0.404	2.464	0.014	0.204	1.788
Br_bin[T.5]	0.716	0.453	1.581	0.114	-0.172	1.605
Br_bin[T.6]	1.740	0.532	3.270	0.001	0.697	2.782
C(WaterType, Treatment(reference='SW'))[T.GU]	-12.555	1.950	-6.438	0.000	-16.377	-8.733
C(WaterType, Treatment(reference='SW'))[T.GW]	-23.206	0.529	-43.846	0.000	-24.243	-22.168
C(WaterType, Treatment(reference='SW'))[T.MX]	-6.065	0.702	-8.634	0.000	-7.442	-4.688
C(Residual, Treatment(reference='CL2'))[T.CLM]	-2.814	0.539	-5.217	0.000	-3.872	-1.757
C(Residual, Treatment(reference='CL2'))[T.ClO2]	-0.334	1.591	-0.210	0.834	-3.452	2.784
C(Residual, Treatment(reference='CL2'))[T.None]	-0.455	0.529	-0.861	0.389	-1.491	0.581
C(Season, Treatment(reference='WIN'))[T.FALL]	1.553	0.207	7.502	0.000	1.148	1.959
C(Season, Treatment(reference='WIN'))[T.SPR]	0.820	0.206	3.978	0.000	0.416	1.224
C(Season, Treatment(reference='WIN'))[T.SUM]	2.003	0.190	10.566	0.000	1.631	2.374
Group Var	189.363	1.050				

C) HAA6Br

Model:	MixedLM	Dependent Variable:	HAA6Br
No. Observations:	8962	Method:	REML
No. Groups:	3164	Scale:	5.6332
Min. group size:	1	Log-Likelihood:	-24721.2875
Max. group size:	9	Converged:	Yes
Mean group size:	2.8		

	Coef.	Std.Err.	z	P>|z|	[0.025	0.975]
Intercept	7.129	0.183	39.035	0.000	6.771	7.487
Br_bin[T.2]	0.580	0.127	4.553	0.000	0.330	0.829
Br_bin[T.3]	1.171	0.146	7.996	0.000	0.884	1.457
Br_bin[T.4]	1.866	0.160	11.658	0.000	1.552	2.180
Br_bin[T.5]	2.677	0.181	14.793	0.000	2.322	3.032
Br_bin[T.6]	4.461	0.215	20.743	0.000	4.040	4.883
C(WaterType, Treatment(reference='SW'))[T.GU]	-1.687	0.765	-2.205	0.027	-3.187	-0.188
C(WaterType, Treatment(reference='SW'))[T.GW]	-4.866	0.207	-23.462	0.000	-5.273	-4.460
C(WaterType, Treatment(reference='SW'))[T.MX]	-0.047	0.275	-0.171	0.864	-0.587	0.493
C(Residual, Treatment(reference='CL2'))[T.CLM]	-1.151	0.212	-5.437	0.000	-1.566	-0.736
C(Residual, Treatment(reference='CL2'))[T.ClO2]	-0.091	0.619	-0.148	0.883	-1.305	1.122
C(Residual, Treatment(reference='CL2'))[T.None]	-0.133	0.206	-0.644	0.520	-0.538	0.272
C(Season, Treatment(reference='WIN'))[T.FALL]	0.210	0.081	2.614	0.009	0.053	0.368
C(Season, Treatment(reference='WIN'))[T.SPR]	0.154	0.080	1.926	0.054	-0.003	0.311
C(Season, Treatment(reference='WIN'))[T.SUM]	0.311	0.074	4.218	0.000	0.166	0.455
Group Var	29.594	0.442				

Table S4 Key: Source water types (WaterType): Surface water (SW), groundwater (GW), groundwater under the influence (GU), and mixed surface and groundwater (MX). Residual types: chlorine (CL2), chloramine (CLM), chlorine dioxide (CL02), and no residual reported (None). Size: Large (L) and small (S). Seasons: winter (WIN), fall (FALL), spring (SPR), and summer (SUM).

Table S5: Limit definitions and performance of logit models for Scenarios 1, 2 and 3; all results are statistically significant (p<0.001).

**Model**<br>*Rationale*	**Simulated limit, MCL or MCLeq (µg/L) used for transformation to binary (0,1)**	**Limit %ile**	**% PWS (x=1 OR y=1)**	**% PWS (x=1 AND y=1) of all y=1**	**True positive sample rate (x=1, y=1)**	**False positive sample rate (x=1, y=0)**	**Probability of x=1 occurring with y=1 (±95% CI)**	
							All data	***Sensitivity analysis: Excluding top 0.02% HAA6Br***
**Scenario 1: HAA5**								
A<br>*Equivalent by %ile*	x: HAA5 > 60.0<br>y: HAA6Br > 27.9	98.4^{th}<br>98.4^{th}	4.5%<br>3.6%	16%	10%	90%	0.10 (0.057–0.17)	0.090 (0.050–0.16)
B<br>*Equivalent by % PWS*	x: HAA5 > 60.0<br>y: HAA6Br > 25.4	98.4^{th}<br>97.8^{th}	4.5%<br>4.5%	14%	7.8%	89%	0.11 (0.060–0.18)	0.094 (0.052–0.16)
**Scenario 2: HAA9**								
A<br>*Equivalent by %ile*	x: HAA9 > 77.0<br>y: HAA6Br > 30.9	98.9^{th}<br>98.9^{th}	3.3%<br>2.6%	29%	22%	78%	0.22 (0.14–0.34)	0.21 (0.13–0.32)
B<br>*Equivalent by % PWS*	x: HAA9 > 77.0<br>y: HAA6Br > 28.5	98.9^{th}<br>98.5^{th}	3.3%<br>3.4%	24%	18%	75%	0.25 (0.16–0.37)	0.24 (0.15–0.36)
C<br>*Equivalent by %ile*	x: HAA9 > 72.0<br>y: HAA6Br > 28.5	98.5^{th}<br>98.5^{th}	4.4%<br>3.4%	34%	24%	76%	0.23 (0.16–0.33)	0.23 (0.16–0.33)
D<br>*Indicator set low*	x: HAA9 > 60.0<br>y: HAA6Br > 28.5	96.5^{th}<br>98.5^{th}	8.8%<br>3.4%	50%	37%	84%	0.16 (0.12–0.21)	
**Scenario 3: Bromide**[a]							***Excluding Br >1 mg/L***	***Sensitivity analysis: All Br data***
A<br>*Equivalent by %ile*	x: bromide > 374<br>y: HAA6Br > 30.2	97.8^{th}<br>97.8^{th}	3.3%<br>2.8%	30%	24%	76%	0.24 (0.16–0.34)	0.18 (0.12–0.28)
B<br>*Equivalent by % PWS*	x: bromide > 374<br>y: HAA6Br > 28.2	97.8^{th}<br>97.3^{th}	3.3%<br>3.4%	26%	21%	74%	0.26 (0.18–0.36)	0.20 (0.13–0.29)
C<br>*Equivalent by %ile*	x: bromide > 314<br>y: HAA6Br > 28.2	96.9^{th}<br>97.3^{th}	4.4%<br>3.4%	28%	26%	77%	0.23 (0.16–0.31)	0.20 (0.14–0.28)
D<br>*Indicator set low*	x: bromide > 200<br>y: HAA6Br > 28.2	93.9^{th}<br>97.3^{th}	8.8%<br>3.4%	49%	42%	81%	0.19 (0.14–0.25)	

a) The dataset for Scenario 3 consists of fewer sample records and PWS, as PWS that did not report bromide were excluded, as were PWFS which reported using membrane filtration were excluded, and bromide records above 1.0 mg/L. The maximum HAA6Br LRAA concentration corresponding to each bromide record was used to avoid repeated measures. The final dataset is 8,647 records from 3,164 PWS.

Table S6: Correlation matrix reporting the rank correlation coefficient (r_s) between each HAA class and bromide concentrations on a continuous basis or binned into the six levels.

**Class**	**Bromide (μg/L)**		**Bromide (binned)**	
	r_s	**p**	r_s	p
**HAA5**	-0.33	1.4E-216	-0.30	7.3E-180
**HAA6Br**	0.25	6.3E-96	0.27	1.6E-74
**HAA9**	-0.22	1.3E-127	-0.19	1.1E-142

Supplementary Figures: S1–S4

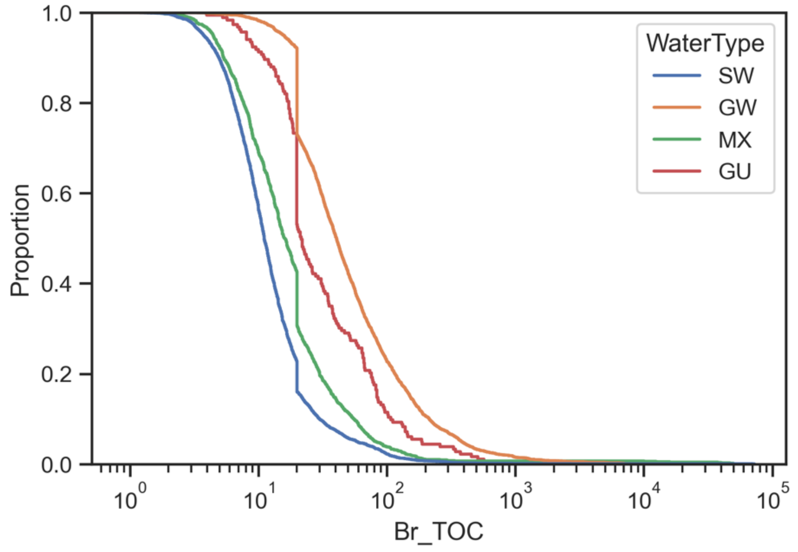

Figure S1: Complementary cumulative density plot showing the distribution of bromide/TOC ratios (µg/L/mg/L) in influent samples from UCMR4 PWS utilizing source waters SW, GW, MX or GU. To avoid issues of repeated measures, bromide and TOC data were averaged by PWS. To avoid division by zero, <MRL values were replaced by MRLs (bromide, 20 µg/L; TOC, 1.0 mg/L).

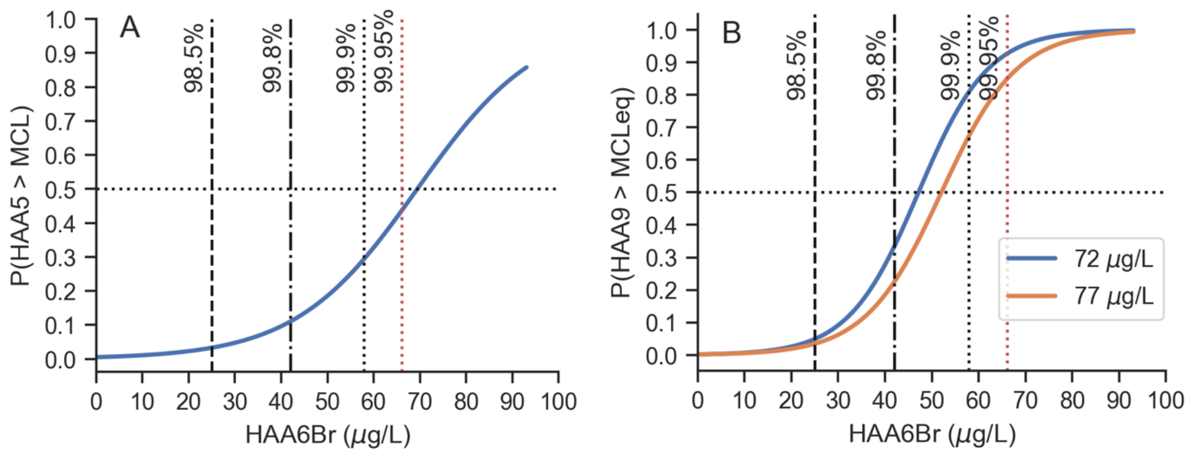

Figure S2: Sensitivity analysis on effect of repeated measures, with LRAA concentrations averaged by PWS for comparison with Figure 1. Probability of an exceedance of an A) HAA5 MCL or B) HAA9 MCL equivalent (MCLeq) of 72 or 77 µg/L co-occurring with any HAA6Br concentration (on an LRAA basis). For visibility, the x-axis was truncated from 165 µg/L (the maximum HAA6Br concentration).

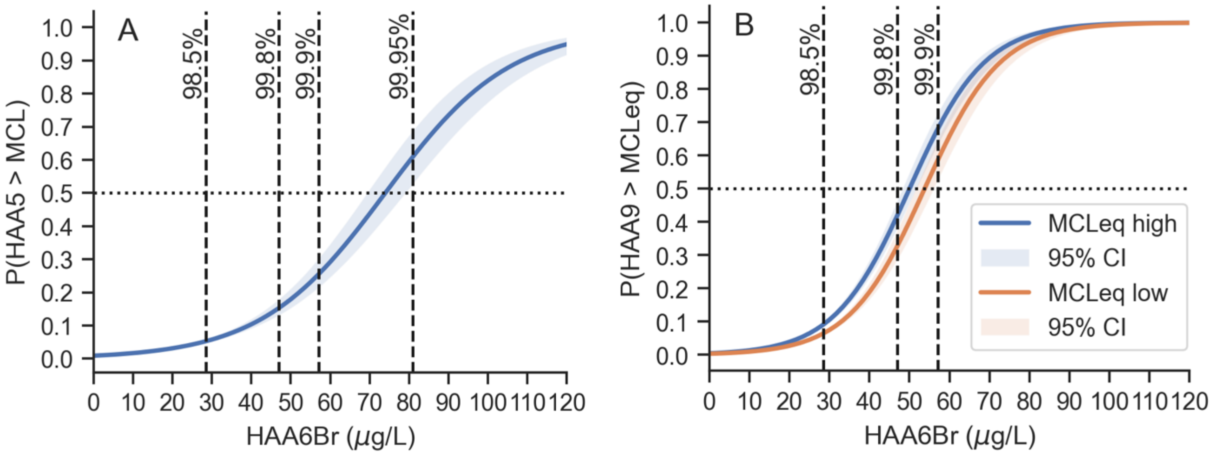

Figure S3: Sensitivity analysis on effect of replacement of Method Reporting Limit (MRL) data, for comparison with Figure 3; the 0.0 µg/L values were not replaced for this figure, whereas the values were replaced with 0.2 µg/L for the analysis in the main text. Probability of an exceedance of an A) HAA5 MCL or B) HAA9 MCL equivalent (MCLeq) of 72 or 77 µg/L co-occurring with any HAA6Br concentration (on an LRAA basis). For visibility, the x-axis was truncated from 165 µg/L (the maximum HAA6Br concentration).

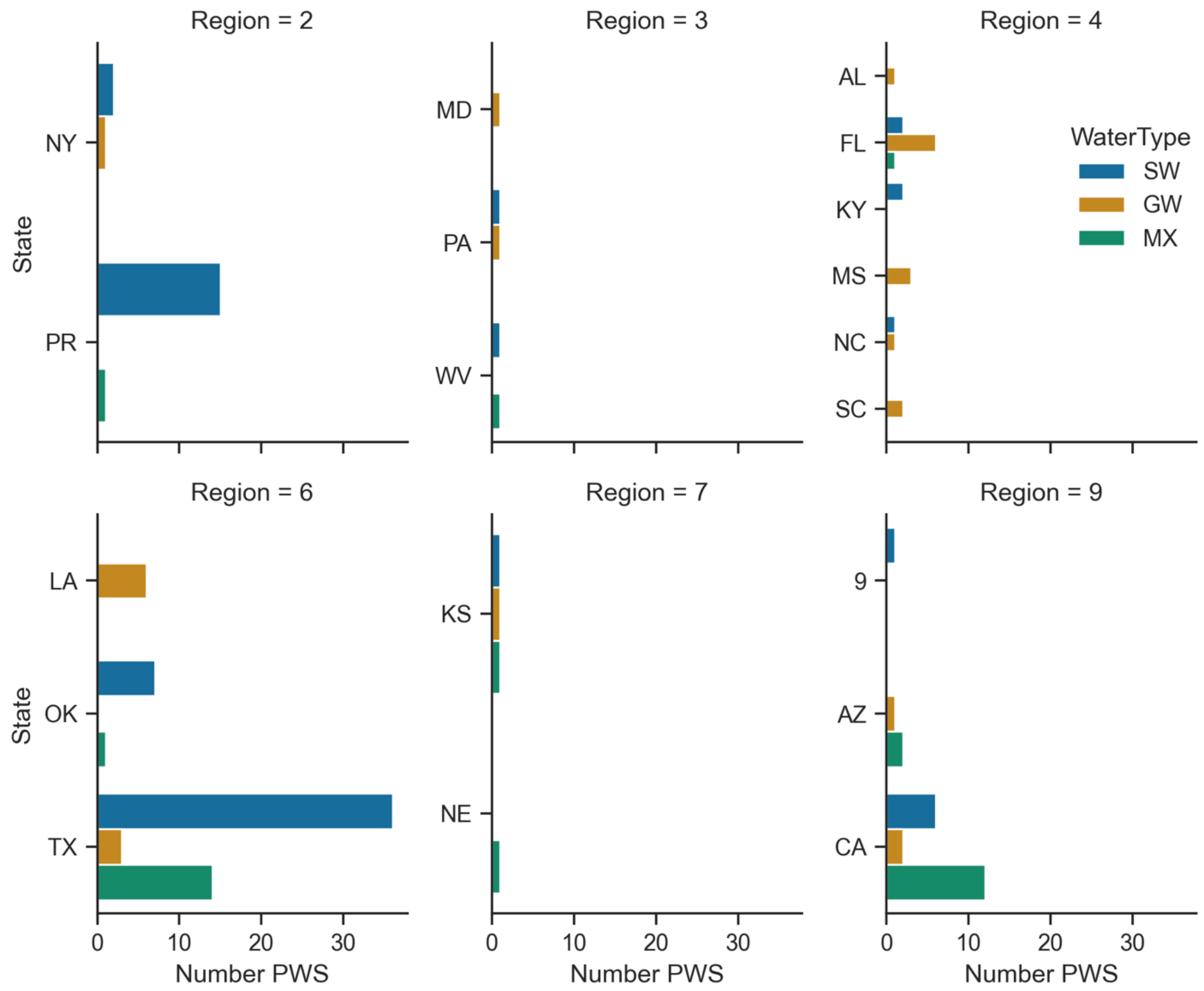

Figure S4: Number of PWS that could be newly implicated by the HAA6Br MCLeq, grouped by EPA Region, State (or EPA Region, for tribal systems), and source water type. EPA Regions 1 and 5 were excluded due to small sample size, while Region 8 has no PWS that may be implicated. In Region 1, PWS that may be implicated consist of one GU system in tribal territory and one GW and one MX system in Massachusetts. In Region 5, Illinois had one SW system that may be implicated.

References

Allaire, M., Wu, H., Lall, U., 2018. National trends in drinking water quality violations. Proceedings of the National Academy of Sciences of the United States of America 115, 2078–2083. https://doi.org/10.1073/pnas.1719805115

City of Chesapeake, n.d. “Plants & Sources.” Accessed May 29th, 2024. Available at: https://www.cityofchesapeake.net/863/Plants-Sources

City of Santa Barbara, n.d. “Water Resources.” Accessed May 29th, 2024. Available at: https://santabarbaraca.gov/government/departments/public-works/water-resources

Furst, K.E., Bolorinos, J., Mitch, W.A., 2021. Use of trihalomethanes as a surrogate for haloacetonitrile exposure introduces misclassification bias. Water Research X 11, 100089. https://doi.org/10.1016/j.wroa.2021.100089

Ged, E.C., Boyer, T.H., 2014. Effect of seawater intrusion on formation of bromine-containing trihalomethanes and haloacetic acids during chlorination. Desalination 345, 85–93. https://doi.org/10.1016/j.desal.2014.04.021

Monterey Water, 2022. “Annual Water Quality Report.” Accessed May 29th, 2024. Available at: https://authoring-dotcms-prod.awapps.com/dA/c9eaad97a6/fileAsset/2022_CCR_Coastal_Monterey_Final+.pdf

Seabold, Skipper, and Josef Perktold, 2010. “statsmodels: Econometric and statistical modeling with python.” Proceedings of the 9th Python in Science Conference.

Statman-Weil, Z., Nanus, L., Wilkinson, N., 2020. Disparities in community water system compliance with the Safe Drinking Water Act. Applied Geography 121. https://doi.org/10.1016/j.apgeog.2020.102264
